# Transabdominal color doppler ultrasonography: A relevant approach for assessment of effects of uterine torsion in buffaloes

**DOI:** 10.14202/vetworld.2016.842-849

**Published:** 2016-08-13

**Authors:** Devender, Ramesh Kumar Chandolia, Anand Kumar Pandey, Vishal Yadav, Parveen Kumar, Jasmer Dalal

**Affiliations:** 1 Department of Veterinary Gynaecology and Obstetrics, Lala Lajpat Rai University of Veterinary and Animal Sciences, Hisar - 125 004, Haryana, India; 2 Teaching Veterinary Clinical Complex, Lala Lajpat Rai University of Veterinary and Animal Sciences, Hisar - 125 004, Haryana, India

**Keywords:** buffaloes, pixel value, transabdominal, ultrasonography, uterine torsion

## Abstract

**Aim:**

The present study was conducted on advanced pregnant buffaloes suffering from uterine torsion to assess the status of fetus and uterus by transabdominal ultrasonography, and the findings were compared with normal advanced pregnant buffaloes.

**Materials and Methods:**

The study was conducted on 20 clinical cases of uterine torsion and 20 normal advanced pregnant buffaloes (control group). The lower ventral area just lateral to linea alba (on both sides of the udder) in standing animals was scanned transabdominally by the two-dimensional convex transducer for various ultrasonographic findings. The data collected were statistically analyzed by “one-way ANOVA” and “independent sample t-test” using computerized SPSS 16.0 software program.

**Results:**

Transabdominal ultrasonography revealed dead fetus in 95% uterine torsion cases and proved useful in imaging internal structures of fetuses while no dead fetus was reported in the control group. Size of umbilicus was found significantly decreased (p<0.05) in uterine torsion group in comparison to control animals, but the decrease in placentomal area was marginal (p>0.05) in uterine torsion group. Average thickness of the uterine wall and mean pixel values of fetal fluids (echogenicity) were found significantly increased (p<0.05) in uterine torsion affected buffaloes in comparison to control group.

**Conclusion:**

Status of fetus (whether live or dead), internal status of uterus, and its contents could be determined by transabdominal ultrasonography in uterine torsion cases and thus determining the prognosis of the uterine torsion cases before going for further manipulations. This will also help in taking all the precautions to avoid death of the fetus.

## Introduction

Torsion of uterus usually occurs in a pregnant uterine horn and is defined as the twisting of the uterus on its longitudinal axis [1,2]. Based on published data, it appears that uterine torsion is the single largest cause of dystocia in buffaloes during terminal gestation [3,4]. It appears to originate because of inherently weaker broad ligaments, smaller quantity of fetal fluids and decrease in uterine tone and size coupled with inordinate fetal movements [3]. Factors such as duration of the condition and severity of the torsion have been suggested as determinants of the outcome [5]. The incidence of uterine torsion, as well as the time of its occurrence in bovines, emphasizes its impact on dam’s health and thus the dairy herd profitability. This includes losses due to calf, reduced milk yield, and handling of subsequent conditions, *viz*., delayed uterine involution, endometritis, and infertility.

At present, there are difficulties in assessing advanced pregnancy with rectal palpation, particularly when the uterus is located in the abdominal cavity. In cases suffering from high degree uterine torsion, it is not possible to palpate the fetus. Up to now, a lot of studies have been carried out regarding biochemical, hematological, and other stress parameters to evaluate the status of fetus and uterus in uterine torsion affected buffaloes. However, a study regarding ultrasonographic assessment of fetus and dam is still lacking. Till now, status of umbilicus, fetal fluids, uterine layer, fetal organs, and viability of fetus has not been assessed by transabdominal ultrasonography. Consequently, there is failure to predict the prognosis of fetus and dam in uterine torsion affected buffaloes, and other advanced tools like ultrasonography might be useful in solving the problem.

The transabdominal ultrasonography has become a widespread ancillary tool for assessing the fetus and its uterine adnexa in many veterinary species [6] and has been studied more extensively in human pregnancies [7]. Although the information available in the bovine pregnancy is not as developed as in human obstetric, interesting information can be obtained by the ultrasonographic assessment of the fetus and the uterine adnexa during the late pregnancy.

## Materials and Methods

### Ethical approval

The study was conducted after the approval of the Institutional Animal Ethics Committee.

### Study area

The study was conducted in the Teaching Veterinary Clinical Complex (TVCC) with the collaboration of the Department of Livestock Production and Management, College of Veterinary Sciences, Lala Lajpat Rai University of Veterinary and Animal Sciences (LUVAS), Hisar (Haryana).

### Animals

The study was conducted on 20 clinical cases of uterine torsion and 20 normal advanced pregnant buffaloes (control group). The animals were admitted to the TVCC with the history of no progress in the parturition process or because of general medical problems such as colic, straining, and reduced feed intake in late pregnant buffaloes (mostly between 8^th^ and 10^th^ month of pregnancy).

### Methodology

The case history for each animal was recorded in history sheet which included the age of the animal, parity, stage of gestation, duration of the condition, and previous intervention and its nature. Diagnosis of uterine torsion was done after careful rectal and vaginal examinations after checking the broad ligament status. Uterine torsion having degree ≤180 were classified as light degree and the one having degree <180 were classified as high degree uterine torsion arbitrarily, solely on the basis of manual judgment.

### Ultrasonographic examination

**Figure-1:**
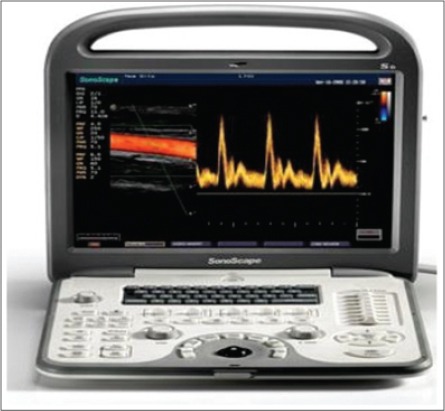
SonoScape S 6 portable digital color Doppler ultrasound machine.

**Figure-2:**
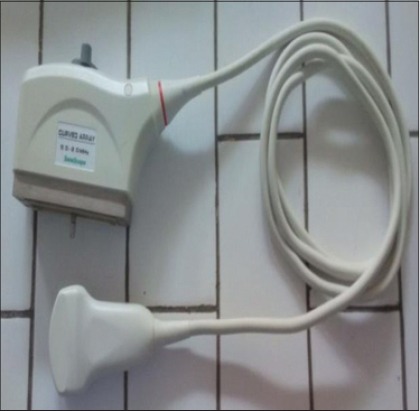
Convex two-dimensional curved array transducer.

A high-quality ultrasound machine (Figure-1; SonoScape S6/S6Pro/S6BW; Portable Digital Color Doppler Ultrasound) equipped with two-dimensional (2D) convex transducer (Figure-2) having switchable frequency between 2.0 and 5.0 MHz designed for transabdominal approach was used for the uterus and fetal ultrasonographic findings. The lower ventral area just lateral to linea alba (on both sides of the udder) was shaved, and coupling gel was applied to take ultrasonographic images. Proper shaving of site helped in proper imaging. From this study, various parameters were recorded like heartbeat of the fetus and fetal internal structures, size and status of umbilicus, size of placentomes, echogenicity of fetal fluid, and thickness of uterine layers. The measurements were plotted in column charts. Pixel values were measured in “Adobe Photoshop” computer software. Minimum 3 to 4 areas for each image were selected to measure the mean pixel values. A particular area of image was selected and then clicked on “edit” and “histogram” to get the mean value.

### Statistical analysis

The ultrasound images recorded in the machine were reviewed in the scanner itself to re-examine the images in detail. The data collected were statistically analyzed for finding out average mean and standard error. Differences at p<0.05 were considered to be statistically significant. One way ANOVA and independent sample t-test were employed using computerized SPSS 16.0 software program.

## Results

As per gestation status, the number of buffaloes with uterine torsions during the 8^th^ month was 10% (2/20), during 9^th^ month was 15% (3/20), and during the tenth month was 75% (15/20). Depending on the case history, the approximate duration of torsion was recorded in 85% buffaloes (17/20). For the remaining 15% (3/20) animals, the duration of torsion depending on clinical symptoms had not been observed exactly by the owner. Torsion was post-cervical with vaginal involvement in 95% (19/20) of the cases. The 5% cases (1/20) were of pre-cervical uterine torsion without vaginal involvement. The degrees of uterine torsion were light and high in 30% (6/20) and 70% (14/20) of the cases, respectively, as per manual and ultrasonographic findings. Right uterine torsion (clockwise) was recorded in 100% (20/20) of the cases. 90% cases (18/20) of the current study were successfully detorted by rolling the dam. Cesarean sections were performed in 10% (2/20) of the cases. The fetal mortality was recorded in 95% (19/20) of cases, and the maternal mortality was reported in 10% (2/20) cases (cases in which cesarean section was performed). All pregnant buffaloes in the control group (n=20) had spontaneous and normal births with viable buffalo calves.

**Figure-3:**
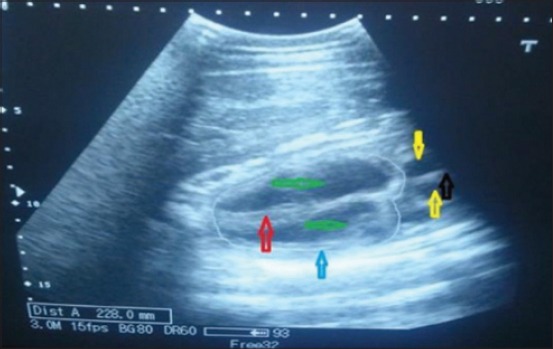
Transabdominal sonographic image of heart of fetus in normal advanced pregnant buffalo. Yellow, green, black, red, and blue arrows represent the lumen of auricles, lumen of ventricles, inter-atrial septum, inter-ventricular septum, and ventricular wall, respectively.

Transabdominal ultrasonography proved very useful in advanced pregnancy and uterine torsion cases where it was very difficult to assess the status of fetus manually. Acoustic shadowing or areas of low-amplitude echoes created by bony structures such as vertebrae and ribs were readily visible in the transabdominal scanning. The fetal heart was observed within the cranial, cone-shaped thorax, with fetal heartbeat being clearly visible in all the animals of the control group (Figure-3). In the current study, the fetus was found dead at the time of presentation in 95% (19/20) of uterine torsion cases, and in 5% (1/20) cases, fetus was live. Fetus was live in 100% cases in the control group. The uterine torsion case, in which fetus was live, its heartbeat recorded was 134 beats per minute (bpm). Fetal heartbeats recorded in normal advanced pregnant buffaloes was 111.90±3.31 bpm (mean±standard error [SE]).

**Figure-4:**
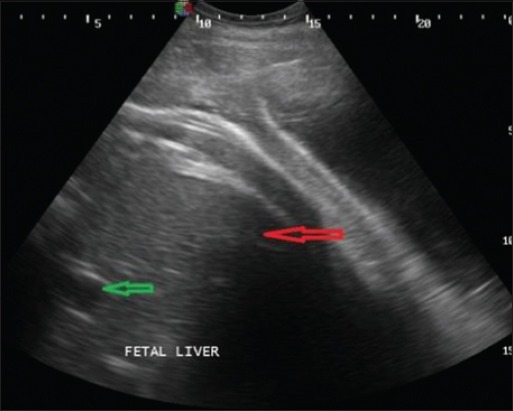
Transabdominal sonographic image of liver of fetus in full term buffalo having right side post-cervical uterine torsion since last 12 h. Liver is shown by red arrow and portal vein is shown by green arrow in the image.

**Figure-5:**
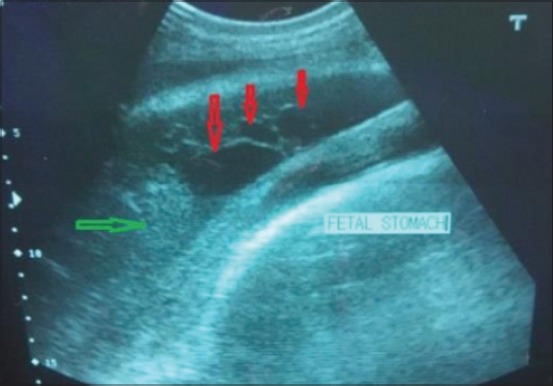
Transabdominal sonographic image of stomach of fetus in normal advanced pregnant buffalo. In the image, ruminal compartments (shown by red arrows) and reticulum (shown by green arrows) are clearly visible.

Transabdominal ultrasonography, using 2.0-5.0 MHz 2D convex transducer produced detailed images of bovine fetal and uterine anatomy. It was observed that fetal structures were easy to image in normal advanced pregnant buffaloes as compared to uterine torsion buffaloes. Still, some fetal structures such as fetal liver (Figure-4), kidney, and heart were successfully imaged in uterine torsion affected buffaloes. The stomach (Figure-5) could be seen as a large, fluid-filled structure, with the fetal reticulum and intestines appearing as echogenic areas.

**Figure-6:**
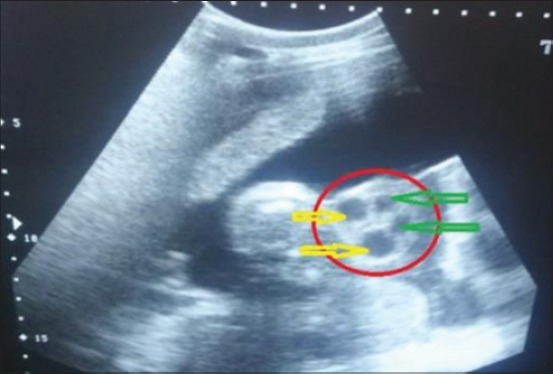
Transabdominal sonographic cross-sectional image of umbilical cord (shown inside the red circle) in normal advanced pregnant buffalo. Two umbilical arteries (marked by yellow arrows) and two umbilical veins (marked by green arrows) are clearly visible in the image.

**Figure-7:**
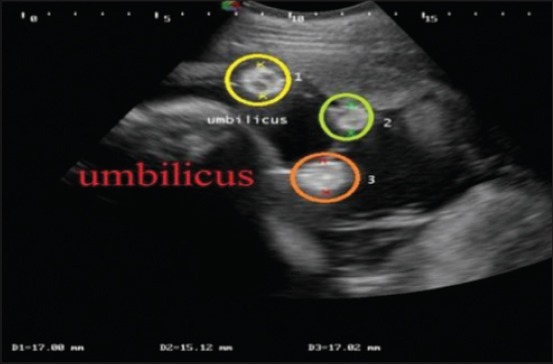
Transabdominal sonographic cross-sectional image of umbilicus in uterine torsion buffalo. In this image, three cross-sections of umbilicus are marked by 1, 2, and 3. Umbilical arteries and veins are not so clearly differentiated.

**Figure-8:**
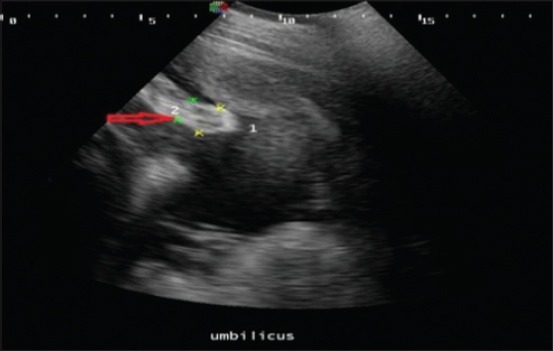
Transabdominal sonographic image of umbilicus (longitudinal view) in uterine torsion buffalo. Umbilicus is shown by red arrow. Umbilical arteries and veins are not so clearly differentiated.

**Figure-9:**
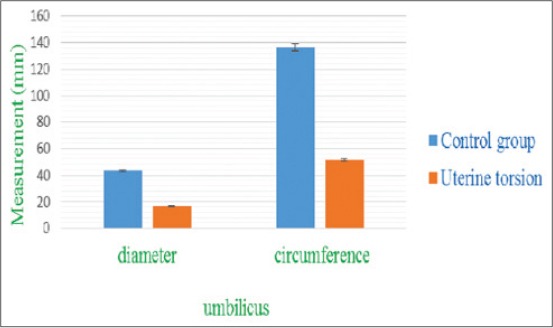
Column chart showing umbilicus diameter and circumference (mean±standard error) in control and uterine torsion affected buffaloes.

**Figure-10:**
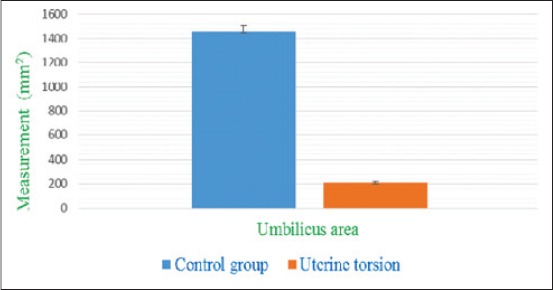
Column chart showing umbilicus area (mean±standard error) in control and uterine torsion affected buffaloes.

The umbilical cord was identified by its characteristic sonographic images showing four echo-poor tubes (cross-section) representing the paired umbilical arteries and veins. In cross-section (Figures-6 and 7), the umbilical vessels were visible in a quadrilateral arrangement, whereas, in the longitudinal view (Figure-8), only one to two vessels were apparent. In uterine torsion cases, umbilical cord (Figure-7) was found suppressed in comparison to normal advanced pregnant buffaloes (Figure-6), and also the umbilical arteries and veins were not so clearly differentiable both in cross-section and longitudinal section. It was observed that the mean±SE values of diameter, circumference (Figure-9), and area (Figure-10) of the umbilicus in uterine torsion buffaloes were significantly lower than the normal advanced pregnant buffaloes (p<0.05). Moreover, umbilical arteries and veins were not so clearly differentiated in uterine torsion affected buffaloes which also might be due to rotation of gravid horn.

**Figure-11:**
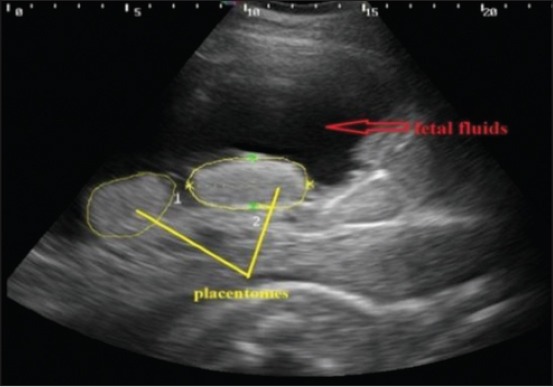
Transabdominal sonographic image of placentomes in normal advanced pregnant buffalo. Two placentomes are visible in the image encircled by yellow circles. Hypoechoic fetal fluid is shown by red arrow.

**Figure-12:**
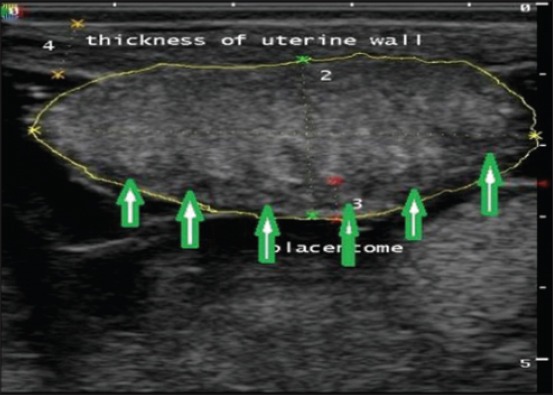
Sonographic image of placentomes in buffalo suffering from uterine torsion. Image showing the changes at the apex of the placentome (toward the uterine lumen) shown by multiple green arrows.

**Figure-13:**
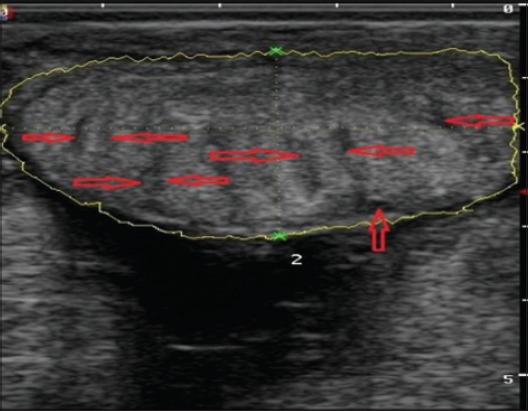
Sonographic image of placentome in buffalo suffering from uterine torsion. Image showing the changes throughout the placentome as depicted by hypoechoic furrows (marked by multiple red arrow).

**Figure-14:**
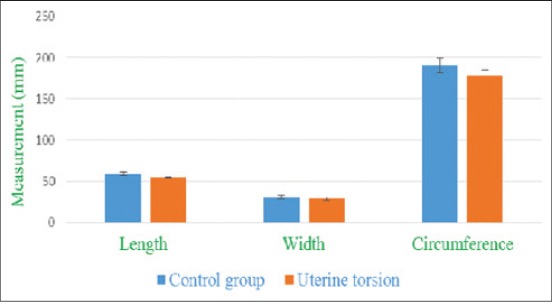
Column chart showing the length, width, and circumference (mean±standard error) of placentomes in control and uterine torsion affected buffaloes.

**Figure-15:**
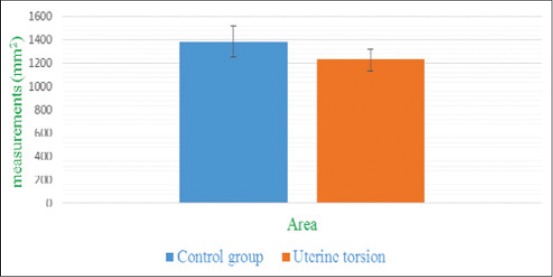
Column chart showing area of placentomes (mean±standard error) in control and uterine torsion affected buffaloes.

The placentomes were easily recognized by their specific aspect of an echogenic ovale to elliptical structure with a dimension of a chicken-egg both in control (Figure-11) and uterine torsion (Figure-12) affected buffaloes. They were distributed on the uterine wall and were observed surrounded by anechoic fetal fluids. The placentomes were well composed, tight together and without any sign of degeneration (Figure-11), in normal advanced pregnant buffaloes. It was interesting to observe details of morphological changes in placentomes either at the apex (Figure-12) or throughout (Figure-13) the placentomes in uterine torsion affected buffaloes. These changes were hypoechoic as compared to the other parenchyma of placentomes. The cotyledonary part of placenta showed fluid accumulation at boundaries of placentomes depicted by hyperechoic areas on corners of cotyledons (Figure-12). In some cases of uterine torsion, there was accumulation of fluid in central part of cotyledons indicating morphological changes occurring in placentomes at the time of placental separation in uterine torsion cases (Figure-13). The size of placentomes (Figures-14 and 15) tended to be lower in uterine torsion cases than normal pregnancy, however, non-significantly (p>0.05).

**Figure-16:**
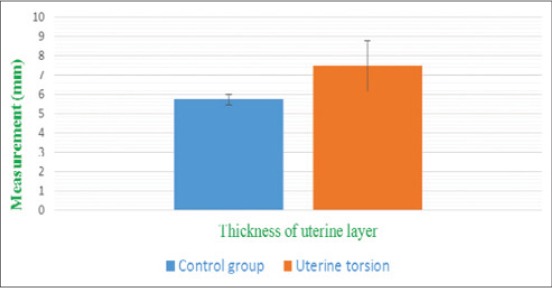
Column chart showing thickness of uterine layer (mean±standard error) in control group and uterine torsion affected buffaloes.

**Figure-17:**
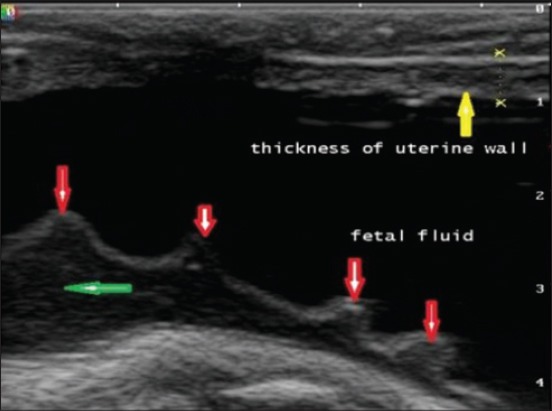
Sonographic image of thickness of uterine wall (marked by yellow arrow) and fetal fluids in normal advanced pregnant buffalo. One peculiar feature of the image was the foldings of amniobiotic membrane (marked by red arrows). Amniotic fluid is shown by green arrow.

**Figure-18:**
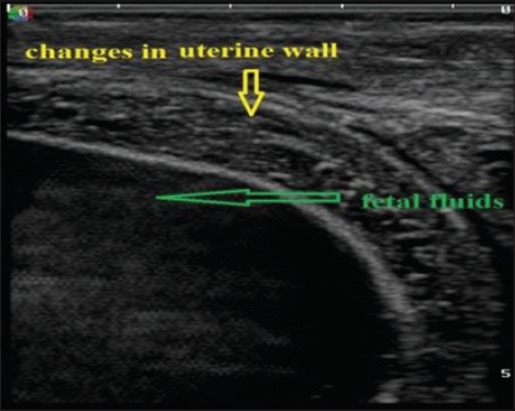
Sonographic image of uterine wall showing inflammatory changes in uterine torsion affected buffaloes.

Uterine layer appeared as a hyperechoic layer over the anechoic fetal fluids in normal and uterine torsion affected buffaloes. A significant increase (p<0.05) in thickness of uterine layer (Figure-16) was found in uterine torsion affected buffaloes as compared to the normal advanced pregnant buffaloes. Moreover, it was also observed that in control group buffaloes, thickness of uterine layer was uniform (Figure-17) throughout but in uterine torsion cases, it was variable at different positions. Inflammatory changes (Figure-18) were observed in uterine wall in long duration cases of uterine torsion. Inflammatory changes were visible as hyperechoic spots in the uterine wall.

**Figure-19:**
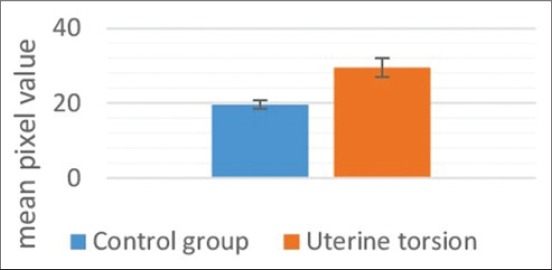
Column chart showing mean pixel values of fetal fluids in control and uterine torsion affected buffaloes.

The amniotic fluid appeared as a hypoechoic fluid with various amounts of echoic particles. Allantoic fluid appeared as an anechoic media. Both uterine fluids were separated by a thin hyperechoic membrane which is the amniotic membrane. In normal advanced pregnant buffaloes (Figure-11), fetal fluids appeared completely anechoic, whereas in uterine torsion (Figure-18) affected buffaloes, fetal fluids have various amount of echoic particles. It was observed that the mean pixel values of fetal fluids in uterine torsion cases (Figure-19) were significantly higher (p<0.05) than the normal advanced pregnant buffaloes.

## Discussion

It appeared that transabdominal ultrasonography was very useful in advanced pregnancy and uterine torsion cases where it was very difficult to assess the status of fetus manually. Status of fetus (whether live or dead) could be ascertained by determining the heartbeat of the fetus by transabdominal ultrasonography. This finding could be useful before further manipulations in uterine torsion cases. This will also help in taking all the precautions to avoid death of the fetus. Jonker *et al*. reported variation of fetal heart rate from 90 to 125 bpm during the last 2 weeks of pregnancy in bovines [8]. Comparable values were also reported by Breukelman *et al*, who reported the mean fetal heart rate varying from 114 bpm in bovine fetuses 3 weeks before birth, to 109 bpm during the last 2 weeks of normal pregnancy [9]. So, the increase in fetal heart beat in uterine torsion in the current study might be due to the decreased blood supply to the uterus, leads to hypoxia, and increased fetal movements which are responsible for tachycardia [8]. Ultrasonography was considered sufficient to assess live and dead status by measuring heartbeat of the fetus, which itself is a confirmatory criterion for assessing the fetal viability. So, the blood samples were not collected in the present study for assessing fetal viability using biochemicals to compare it with ultrasonographic findings.

The accessibility of various organs and parts of the fetal body depended on the orientation of transducer with respect to fetus and position of the fetus. In the past, various frequencies have been used for transabdominal ultrasonography [10]. In the present study, transabdominal ultrasonography using 2.0-5.0 MHz 2D convex transducer produced detailed images of bovine fetal and uterine anatomy, and these images were of quality that was not dissimilar to those which have been reported for smaller species. Therefore, it is inferred that this frequency is very useful in late pregnancy and uterine torsion cases. This type of probe was used for transabdominal ultrasonography because it penetrates further into soft tissues. Although, it results in lower tissue resolution relative to 7.5 MHz probe which is commonly used in transrectal ultrasonography. The relatively small surface area and arc-shaped beam of convex transducers, in comparison with linear transducers, enhance their use in transabdominal ultrasound with easy skin positioning and a wide field of view [11]. Furthermore, many of the fetal structures that were difficult to visualize via transrectal ultrasonography were readily accessible by transabdominal ultrasonography. It was observed that fetal structures were easy to image in normal advanced pregnant buffaloes as compared to uterine torsion buffaloes that might be due to rotation of fetus inside the gravid horn leading to change in position of the fetus in uterine torsion. Altered position of fetus with the majority in dorsoilial (17%) or dorsopubic (43%) was also reported previously in uterine torsion-affected bovines [12]. The ultrasound beams were unable to reach the required depth which might be due to the fact that ultrasound beams were unable to penetrate the dead fetus. Still, some fetal structures such as fetal liver, kidney, and heart were successfully imaged in uterine torsion affected buffaloes in the current study, which might be possibly due to light degree uterine torsion, in which change in position of fetus was not so high as were in higher degree uterine torsion cases. Therefore, transabdominal ultrasonography in the current study appeared very-very useful to assess antenatal fetal organs and their status. Previously, the transabdominal ultrasonography has also proved helpful in two Holstein cows (with prolonged gestation) in determining the pathologic findings in fetuses [13].

The decrease in umbilical cord measurements in uterine torsion cases might be due to stretching of umbilicus or pressure of gravid horn (due to rotation) on the umbilical cord. This decrease in diameter of umbilical cord may also be attributed probably due to decreased blood supply in umbilical arteries and veins in cases of uterine torsion. Moreover, umbilical arteries and veins were not so clearly differentiated in uterine torsion affected buffaloes which also might be due to rotation of gravid horn. Since no previous ultrasonographic studies related to umbilicus measurements were available for comparison with the findings of the current study in uterine torsion affected buffaloes. So, the current study could be useful for reference values of umbilicus measurements in uterine torsion affected buffaloes.

In the present study, morphological changes might be either due to inflammatory or degenerative changes in placentomes or due to separation of placenta in long duration uterine torsion cases. Since there are no parallel studies regarding the ultrasonographic evaluation of morphological changes in placentomes in uterine torsion affected buffaloes for comparison of these findings. So, the ultrasonographic findings in the present study could be useful as a reference study for the upcoming studies in uterine torsion affected buffaloes and could prove helpful in determining the status of buffaloes at the time of presentation of clinical cases of uterine torsion. This marginal reduction in placentomal area might be due to the necrobiotic changes and infarction which originated from the blood supply compression and obstruction. These findings are comparable to the findings of Hussein (2013), who also reported the reduction in placentomes size in long-standing cases of uterine torsion [14]. The measurement of placentomal size could be a useful asset in diagnosing the abnormal pregnancies as the study carried by Buczinski *et al*. determined the pathological findings in the fetuses in two Holstein cows in prolonged gestation by transabdominal ultrasonography and measured the mean placentomal area 29.1 cm^2^ which was higher than the normal placentomal measurements of advanced pregnant cows [13]. This increased placentomal area in cows in study by Buczinski *et al*. might be because of prolonged gestation of cows [13].

The current study showed a significant increase in the thickness of uterine wall which might be due to limited arterial perfusion and venous outflow in the twisted uterus which leads to ischemia, hypoxia, and cell death causing irreversible damage to the endometrium and myometrium. As the obstruction prolonged, inflammation progresses, and bacterial infection can spread to placenta and uterine wall. However, there are no parallel studies regarding ultrasonographic measurements of uterine wall thickness in uterine torsion affected buffaloes. So, the present study could be useful as a reference study regarding changes in uterine wall in uterine torsion cases.

Compared to the allantoic fluid, the echogenicity of the amniotic fluid was higher in normal as well as in uterine torsion affected buffaloes during advanced gestation. This was reflected in higher mean pixel values. In the current study, pixel values (echogenicity) of fetal fluids were found increased in uterine torsion affected buffaloes. Previously, increase in echogenicity of fetal fluid in mare has been attributed to the passage of meconium in utero, hemorrhage, or inflammatory debris and that might reflect fetal hypoxia, placental detachment, or placenta1 infection [15]. So, the increase in echogenicity in the present study was comparable to the study done by other workers previously in different species.

## Conclusion

Whenever a case of uterine torsion is presented, it is of utmost importance for a veterinarian to know the status of the fetus (whether live or dead) before further manipulations for the well-being of both the dam and the calf. Status of the fetus can be easily determined by transabdominal ultrasonography by observing the fetal heart beats. This finding could be useful before further manipulations in uterine torsion cases. This will also help in taking all the precautions to avoid death of the fetus. From this study, it was concluded that it is comparatively easier to image the internal structures of the fetus in normal advanced pregnant buffaloes as compared to uterine torsion affected buffaloes. Umbilicus measurements decreased in buffaloes having uterine torsion. It could be possible to visualize morphological changes in placentomes and inflammatory changes in uterine wall by ultrasonography in uterine torsion affected buffaloes. By assessing the ultrasonographic images of fetal fluids for pixel values, it could be possible to assess the clinical case of uterine torsion in more efficient manner.

## Authors’ Contributions

DD, RKC, and AKP proposed the study. DD, VY, and PK carried out the ultrasonography and management of the plan of study. DD and JD critically observed the data and calculated mean and used SPSS statistical software. DD, PK, and VY prepared the manuscript. Finalization of the manuscript was done by RKC, AKP, and DD. All authors read and approved the final manuscript.
